# Airway Administration of Bacterial Lysate OM-85 Protects Mice Against Respiratory Syncytial Virus Infection

**DOI:** 10.3389/fimmu.2022.867022

**Published:** 2022-05-05

**Authors:** Krist Helen Antunes, Gisele Cassão, Leonardo Duarte Santos, Sofia Giacomet Borges, Juliana Poppe, João Budelon Gonçalves, Eduarda da Silva Nunes, Guilherme Fernando Recacho, Vitória Barbosa Sousa, Gabriela Souza Da Silva, Daniel Mansur, Renato T. Stein, Christian Pasquali, Ana Paula Duarte De Souza

**Affiliations:** ^1^ Laboratory of Clinical and Experimental Immunology, School of Health and Life Science, Pontifical Catholic University of Rio Grande do Sul (PUCRS), Porto Alegre, Brazil; ^2^ Laboratory of Imunobiology, Department of Microbiology, Immunology and Parasitology, Universidade Federal de Santa Catarina, Florianópolis, Brazil; ^3^ Department of Pediatrics, São Lucas Hospital PUCRS, School of Medicine, Pontifical Catholic University of Rio Grande do Sul (PUCRS), Porto Alegre, Brazil; ^4^ OM Pharma SA, Department of Preclinical Research, Meyrin, Switzerland

**Keywords:** respiratory syncytial virus, OM-85, postbiotic, antiviral response, lung microbiota

## Abstract

Respiratory syncytial virus (RSV) is a seasonal pathogen responsible for the highest percentage of viral bronchiolitis in pediatric patients. There are currently no vaccine available and therapeutic methods to mitigate the severity of RSV bronchiolitis are limited. OM-85, an oral standardized bacterial lysate isolated from human respiratory strains and widely used to prevent recurrent infections and/or exacerbations in populations at risk, has been shown to be effective and safe in children and adults. Here, we demonstrate that airway administration of OM-85 in Balb/c mice prior to infection prevents RSV-induced disease, resulting in inhibition of viral replication associated with less perivascular and peribronchial inflammation in the lungs. These protective effects are dose and time-dependent with complete protection using 1mg dose of OM-85 only four times intranasally. Mechanistic insights using this topical route in the airways revealed increased alveolar macrophages, a selective set of tolerogenic DCs, Treg and Th1 expansion in the lung, even in the absence of infection, contributing to a better Th1/Th2 balance and preventing ILC2 recruitment in the airways and associated inflammatory sequelae. OM-85 preventive treatment also improved antiviral response by increasing IFNβ and its responsive genes in the lung. *In vitro*, OM-85 protects against RSV infection in a type I interferon pathway. Our animal model data suggest that intranasal use of OM-85 should be considered as a potential prophylactic product to prevent RSV bronchiolitis once human studies confirm these findings.

## Introduction

Acute viral bronchiolitis caused by RSV is characterized by a highly inflammatory condition of the airways, becoming one of the most prevalent diseases in children in the first two years of life (1), causing approximately 190,000 deaths per year worldwide (2). Hospitalization costs for children with RSV bronchiolitis in rich countries exceed US$ 500 million annually (3, 4), and in middle-income tropical countries costs are around US$ 196 million (5). Still to date, there are no vaccines against the virus, even though new candidates have been extensively studied and are closer to being commercialized (4, 6). Currently, the only available preventive intervention is the humanized anti-RSV F Protein monoclonal antibody (Palivizumab). However, in addition to being expensive, this antibody was shown to reduce hospitalizations and clinical severity of RSV infection in 55% of high-risk children in a randomized controlled clinical trial (7). Thus, interventions to decrease viral replication and consequent reduction in disease severity have received great attention from researchers in the field (8, 9).

The COVID-19 pandemic changed the landscape of almost all other viral respiratory infections leading transiently to a major decrease of viral cases including a delayed RSV outbreak (10). Public health and restrictive measures targeted at controlling transmission of SARS-CoV-2 have altered the epidemiology and seasonality of many respiratory viruses (11–13). For instance, social distancing and sanitary control measures regarding COVID-19, such as the use of face masks, were associated with a 98% reduction in RSV detections through winter 2020 (13, 14). As restriction measures have been eased resulting from the control of the pandemic, there has been an unprecedent resurgence of RSV from the 2020 summer onwards (15–17). These offseason peaks of RSV potentially present a threat to susceptible infants, making the quest for novel and accessible preventive interventions centerstage.

Several studies indicate that the gut microbiota has relevant effects on the clinical course and severity outcome of respiratory infections (18–21). Preclinical data demonstrate that a high-fiber diet protects animals against RSV infection through the modulation of the intestinal microbiota and consequent induction of the production of short-chain fatty acids, mainly sodium acetate (18). Treatment performed alone with acetate was also shown to protect against RSV, reducing viral load and inflammatory infiltrate in the lungs, and this effect was mediated through the increase in the main antiviral molecule, type 1 interferon (IFN-β) and its responsive genes (ISGs) (18, 20). Similarly, respiratory microbiota has also an important role impacting on the mucosal immune phenotype (22–24). Lung microbiota composition is different from the gut, *Haemophilus* spp.*, Pseudomonas* spp.*, Streptococcus* spp., and *Veillonella* spp. are frequently found in the airways and not in the gut (25). Alteration on the lung microbiota have been described in many respiratory diseases (26, 27); also, the use of lung microbiota components and administration direct into the airways have been proposed to be protective against respiratory infections (28, 29). Very recently our group showed that acetate treatment delivered directly into the airways protects against RSV infection through improvement of antiviral response modulated by RIG-I (30). Considering acetate is not a drug used in human, in the present study, we asked if a commercial product currently used orally to prevent recurrent RTIs could confer identical protection against RSV.

To this end, we used OM-85 (Broncho-Vaxom™), a bacterial lysate used in the last 40 years in children and adults and with a good safety profile. Among the extensive literature existing on OM-85, it was shown to induce type I interferon and anti-inflammatory responses through different immune mechanisms and effectors cells involving dendritic cells, macrophages, B and T cells (30). Manufactured from 21 respiratory bacteria originally isolated from human, its composition comprises of a mixture of *H. influenzae, S. pneumoniae, K. pneumoniae, Klebsiella ozaenae, S. aureus, Streptococcus pyogenes, Streptococcus viridans, and M. catarrhalis.* According to the international Scientific association of probiotics and Prebiotics, OM-85 can be considerate a postbiotic since it is a preparation of inanimate microorganisms and/or their components that confers a health benefit on the host (31).

One of the mechanisms suggested for the protection of OM-85 against viral infections is the induction of an antiviral response mediated by type 1 interferon (32). However, OM-85 can also modulate the immune response in different ways. It can stimulate expression of beta defensin-1 from primary human lung airway cells as well as C1qRp (CD93) which increases IFN-gamma secretion and CD40 expression on dendritic cells, thereby fighting viral infection and associated secondary bacterial infections. Furthermore, one of the immunomodulatory effects of OM-85 is the regulation of T cells, mainly through the prevention/reduction of Th2-type cytokines and associated inflammation, favoring Th1 cytokines and Treg cells (33–36). While these immunomodulatory effects of OM-85 were seen when the treatment was given orally or directly on airway cells; recent data showed that intranasally administrated OM-85 can confer stronger protection against several models of experimental asthma by targeting dendritic cells and the epithelium/IL-33/ILC2 axis (37).

With our previous work of an intranasal preparation of microbiota-derived acetate protecting against RSV (30), we hypothesized that several constituents from the microbial content from OM-85 could similarly mimic such protective effects against RSV. Similar protective effect with oral OM-85 were observed with *in vivo* studies against H1N1 and betacoronavirus, *in vitro* with human rhinovirus (hRV) infected lung primary epithelial cells, by reducing either SARS-CoV-2 binding proteins on epithelial cells from endobronchial biopsies, and also in live SARS-CoV-2 infection using Vero cells (38, 39). In this study we thus aimed to investigate the effects of intranasal administration of OM-85 and its potential protective effect in RSV infected mice.

## Material And Methods

### Virus and Cell Culture

RSV A2 strain (by kind donation of Dr. Fernando Polack, Fundación Infant, Argentina) was produced in Vero cells grown in Opti-MEM medium supplemented with 2% FBS at 37°C under 5% CO_2_. To assess viral titers Vero cells were infected with RSV in serum-free medium followed by a carboxymethylcellulose plaque assay. Plaque lysis titration were performed using an anti-RSV antibody (Millipore Cat# AB1128, RRID: AB_90477) and viral titers are expressed in plaque forming units (PFU). Virus aliquots were stored at -80°C until the experiment. Mycoplasma-free wild-type, *RIGI* or *IFNAR1* knockout A549 cells generated by CRISPR-Cas9 (ATCC Cat# CCL-185, RRID: CVCL_0023) were cultured in DMEM/F12 medium supplemented with 10% FBS at 37°C under 5% CO_2._

### Mice

SPF-female BALB/c mice (6–8-week old) were bred at the Center for Experimental Biological Models (CeMBE/PUCRS). All animal procedures were performed in accordance with protocols approved by CEUA/PUCRS (protocol #10180). The housing room was set to a 12 h light/dark cycle with lights off at 8 a.m., a temperature of about 22°C and a relative air humidity of about 50%. Mice received sterile drinking water and food in an *ad libitum* regimen. All the animals were monitored daily (Grimace scale was used to measure pain in mice), and at the end of the experiment the animals were euthanized with a lethal dose of anaesthetic through intraperitoneal route. ARRIVE guidelines’ (Animal Research: Reporting of *In Vivo* Experiments) checklist was followed during the planning, conduction, and writing of the study.

### *In Vivo* Treatment and Infection

Unless indicated differently, mice were anesthetized with 5% isoflurane and received 1 mg of OM-85 active principal ingredient (API) (corresponding to 40 μL of OM-85 concentrate, 20 μL/nostril) or saline solution (referred to as “PBS”) *via* the intranasal route. The treatment was performed in 4 different approaches: 1) starting 12 h after infection and given daily throughout 5 days post infection; 2) starting 1 day prior to infection (1 puff); 3) starting 3 days prior to infection (3 puffs); 4) starting 5 days prior to infection (4 puffs). For RSV infection, at the respective time, mice were once again put under light anesthesia, and infected intranasally with RSV strain A2 (10^7^ PFU/ml) in 50 μL of saline solution. All animals were weighed daily, and data analysis performed on day 5 post-infection. We kept 5 and 3 animals per cage for each group (total of 8 animals/per group in each experiment). The animals were randomly allocated in each group.

### Bronchoalveolar Lavage (BAL)

After an overdose of intraperitoneal administration of ketamine solution (0.4 mg/g) and xylazine (0.2 mg/g), mice had their trachea exposed and cannulated with a 14G needle and 5 ml syringe. The lungs were washed twice with PBS 1X 2% FBS. The BAL was centrifuged, and the supernatant was collected for cytokine analysis. The cell pellet was suspended for total and differential cell count and flow cytometry. For differential cell count cell suspension was placed onto to a cytospin funnel and spun down onto Shandon cytoslide at 800 rpm for 5 min using the Cytospin3 system (Shandon, USA). Cytospin slides were stained with rapid panoptic solutions (Laborclin, Brazil) and the counting procedure was performed blindly by an experienced operator using the microscope. Percentages of cell population (macrophages, neutrophils, eosinophils, and lymphocytes) were determined within a total population of 200 cells.

### Histopathological Analysis

The left lung was harvested, fixed in 10% formalin, and embedded in paraffin blocks. The blocks were cut into 4 μm sections, stained with hematoxylin and eosin (H&E). The inflammatory infiltrate was measured in micrometers, 10 measurements were performed starting from the end of the bronchial or vessel epithelium to the end of the inflammatory infiltrate using Olympus CellSens Standart software. Slide analysis was performed blindly. Peribronchial and perivascular inflammation were scored as absent (0), minimal (1), slight (2), moderate (3), marked (4), or severe (5).

### Real-Time PCR of Lung Tissue

Total lung RNA was extracted using TRIzol^®^ reagent (Thermo Fisher Scientific, Waltham, MA, USA) following the manufacturer’s instructions. Complementary DNA (cDNA) was synthesized from the extracted total RNA using the GoScript^®^ Reverse Transcription Kit (Promega^®^, Madison, WI, USA). cDNA samples were stored at -20°C until PCR analysis. Real-time PCR analyses were performed using 15 ɳg of cDNA. The quality of the cDNA for the sample was tested by endogenous β-actin and GAPDH gene amplification using TaqMan-specific primers and probes (Mm02619580_g1 *Actb*, Mm99999915_g1 *Gapdh*, Thermo Fisher Scientific). The TNFa, IL-1β, IFNγ, IL-4, IL-6, IL-8, IL-10, IFNβ, ISG15 and RIG-I expression was measured using TaqMan Assay (complete table describing the gene ID is available on Supplementary data). PCR conditions followed the TaqMan™ Universal PCR Master Mix (Thermo Fisher Scientific, Waltham, MA, USA) protocols. Quantitation of gene expression was conducted using StepOne™ (Applied Biosystems). ΔΔCt or 2^-ΔCt^ were used to calculate gene expression.

### Virus Quantification

The RSV viral load in the lung was accessed by RSV F protein gene expression using the indicated specific primers and probes: forward primer 5’-AACAGATGTAAGCAGCTCCGTTATC-3’, reverse primer 5’-GATTTTTATTGGATGCTGTACATTT-3’, and probe 5’FAM/TGCCATAGCATGACACAATGGCTCCT-TAMRA/-3’. Primer sequences were synthesized and cloned into pUC57 plasmids (GenScript, Piscataway, NJ, USA), to perform a 10-fold dilution to generate a standard curve for viral load calculation. The values obtained from viral copies (based on concentration of the plasmid control) were calculated relative to the weight of the pre-weighed lung portion of 100 mg (copies per gram of lung). To access the replicative RSV particles, the right lungs were homogenized, centrifuged and the supernatant were collected, then inoculated onto Vero cell monolayers. Viral PFUs were determined using an anti-RSV (1:500, Millipore Cat# AB1128, RRID: AB_90477) antibody after 4 days.

### Cytokine Production

The levels of TNFα, IL-1β, IFN-β, IL-4, and IL-10 were quantified in the lung homogenate supernatants by ELISA assay (R&D Systems). The assays were performed according to the manufacturer’s instructions and read at 490 nm wavelength, using an EZ Leia 400 microplate reader (Biochrom).

### Flow Cytometry

Cells were isolated from the lung by perfusion with PBS and then digested in RPMI 1640 medium supplemented with 1 mg/ml of Collagenase IV (Invitrogen-Gibco). Cells isolated from the lung and BAL were incubated with Mouse Fc Block (#553141 BD Biosciences^®^) for 20 min and then stained with LIVE/DEAD™ Fixable Violet Dead Cell dye for 20 minutes. Cells were washed and stained with surface antibodies anti-Lineage Cocktail (1:200, #133303), anti-CD45 (1:200, #557235, clone 30-F11), anti-CD4 (1:200, #100408, clone GK1.5), anti-CD3 (1:200, #100236, clone 17A2), anti-IL-33R (ST2) (1:100, #46-9333-82, clone RMST2-33), anti-CD90.2 (Thy-1.2) (1:100, #17-0902-82, clone 53-2.1), anti-CD11b (1:200, #101206, clone M1/70), anti-CD64 (1:100, #139314, clone X54-5/7.1), anti-F4/80 (1:100, #123126, clone BM8), anti-CD170 (Siglec-F) (1:100, #155508, clone S17007L), anti-CD11c (1:100, #561241, clone HL3), anti- I-Ad/I-Ed (1:200, #558593, clone 2G9), anti-CD25 (1:100, #552880, PC61) (BD Biosciences^®^ and BioLegend), anti-CD207 (langerin) FITC (1:100, #14-2073-82, clone eBioRMUL.2), CD103 PE-Cy7 (1:200, #121425, clone 2E7) (eBioscience and BioLegend). For intracellular staining, cells were fixed with cytofix/cytoperm (BDBisciences^®^) and stained with anti-IL-4 (1:50, #562045, clone 11B11) (BD Biosciences^®^), anti-FoxP3 (1:50, #72-5775, clone FJK-16s), anti-IFNγ (1:50, #RM9001, clone XMG1.2) (eBioscience) and anti-IL-Gata3 (1:50, #25-9966-42, clone TWAJ) (eBioscience) antibodies. The samples were acquired in the flow cytometer BD FACS Canto-II (BD Biosciences^®^). Data were analyzed in FlowJo software (version 10, Tree Star Inc., MA, USA). Gating strategy for each panel is available on **Figure S1**.

### *In Vitro* Analysis

A549 WT, A549 *IFNAR1* ^-/-^, and *RIG-I* ^-/-^ were pre-treated with 7.5 ug/ml OM-85 for 24 hours in the presence of DMEM/F12 10% FBS. After that, the medium was replaced with new DMEM F12 5% FBS and the cells were infected with 10^4^ PFU/ml of RSV A2. Cells were harvested and processed for RNA extraction and cDNA synthesis 24 hours after infection to access the expression of IFN-β genes and ISGs (ISG15, MDA-5 (*IFIH1*) and RIG-I (*DDX58*)). Cell death and viability were measured by MTT assay, and propidium iodide (PI) by flow cytometry; viral load was assessed by real-time PCR 96 hours after infection.

### Immunofluorescence Analysis

Cells were fixed with PBS 1X 0.5% Triton X-100 for 20 minutes and incubated with Fc Block (supernatant of 2.4G2 cells with 5% heat-inactivated human serum) for an additional 20 minutes. Cells were stained with 1:500 of goat anti-RSV antibody (Millipore Cat# AB1128, RRID: AB_90477) followed by 1:1000 of secondary rabbit anti-goat IgG (H + L)-HRP (Santa Cruz Biotechnology Cat# sc-2922, RRID: AB_656965) antibody. Antibody dilutions and washes were performed in 1X PBS. Nuclear staining was performed with Hoechst 33342 (ThermoFisher Scientific), and slides were mounted with glycerin for analysis under a fluorescence microscope (Olympus). Images were acquired using CellSens software (Olympus). Fluorescence intensity and quantification were measured using ImageJ.

### Real-Time PCR of *In Vitro* Analysis

Complementary DNA (cDNA) was synthesized from the extracted total RNA using the GoScript^®^ Reverse Transcription Kit (Promega^®^, Madison, WI, USA). cDNA samples were stored at -20°C until PCR analysis. Real-time PCR analyses were performed using 15 ɳg of a cDNA sample. The quality of the cDNA for each sample was tested by endogenous β-actin gene amplification using TaqMan-specific primers and probes (Hs01060665_g1 ACTB, Thermo Fisher Scientific). The RSV viral load was accessed by RSV F protein gene expression using the indicated specific primers and probes as described above. IFNβ, ISG15, MDA5, and RIG-I expression was measured using TaqMan Assay (complete table describing the gene ID is available in Supplementary data). PCR conditions followed TaqMan™ Universal PCR Master Mix (Thermo Fisher Scientific, Waltham, MA, USA) protocols. Quantitation of gene expression was conducted using StepOne™ (Applied Biosystems). 2^- ΔCt^ were used to calculate gene expression.

### Statistical Analysis

The *in vivo* experiments were performed using 8-14 mice per group. The *in vitro* experiments were carried out in quintuplicate and repeated at least three times. The results obtained were analyzed in the GraphPad Prism 8 statistical software package. One-way ANOVA and Kruskal-Wallis were used for multiple comparisons (with Dunn’s multiple comparison test as *post hoc*). When comparing two different non-parametric groups, Mann-Whitney test was used. The significance level was set at p ≤ 0.05.

## Results

### Therapeutic OM-85 Treatment Does Not Protect Against RSV-Induced Disease

**Figure 1:**
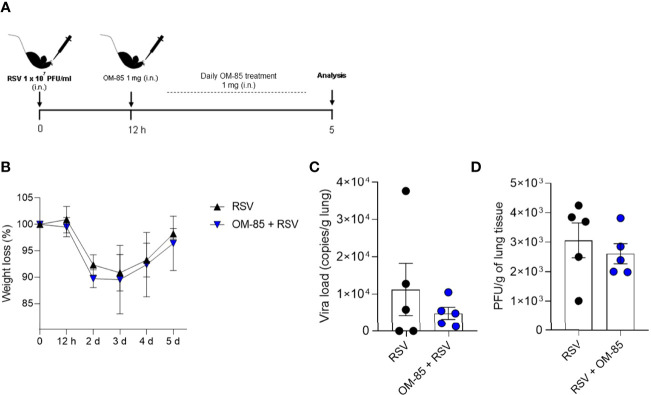
Therapeutic treatment with OM–85 does not modulate antiviral response against RSV infection. Mice were infected intranasally with RSV (1x10^7^ PFU/ml) and 12 hours later started to receive intranasal administration of OM–85 (1mg). Treatment persisted daily until 5 days post infection. **(A)** Experimental design. **(B)** Percentage of weight loss relative to day 0 (right before infection). **(C)** RSV viral load detected in lung tissue by real–time PCR (viral copies/g of lung tissue). **(D)** Viral titer in the lung (PFUs/g lung tissue). Data from one independent experiment (n = 6 per group). All data are expressed as mean ± SEM. Statistical significance between groups was determined by Mann–Whitney. A *p* value < 0.05 was considered significative.

We have recently showed a significant protective effect of the short-chain fatty acid acetate, a microbiota metabolite, in inducing an antiviral response against RSV infection when administered intranasally (18, 30). OM-85 is a bacterial lysate, and we sought to investigate the same therapeutic approach and route using this microbial product during RSV-induced disease. To determine whether OM-85 treatment modulates physiological response against RSV infection, mice were inoculated with RSV and after a period of 12 hours were treated with 1 mg of OM-85 *via* intranasal route, similar to the protocols used in asthma models (37, 39). The treatment was administered daily until day 5 post-infection, which is usually the peak of the replication in the experimental models of RSV infection (**Figure 1A**). Different from what we found with acetate (18, 30), OM-85 treatment did not interfere in weight loss caused by RSV-induced disease (**Figure 1B**), nor any difference in cell content in the BAL (36,87% of macrophages in RSV group, and 38,1% of macrophages in RSV + OM-85 group). We also did not find differences in lung viral loads (**Figures 1C, D**), suggesting that therapeutic treatment with OM-85 does not modify outcomes of established RSV infection as in this model.

### Preventive OM-85 Treatment Protects Against RSV Infection in a Time-Dependent Manner

**Figure 2:**
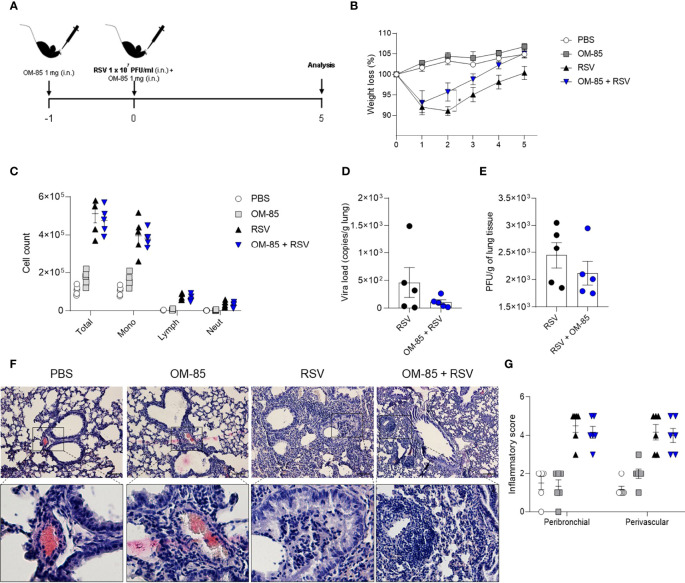
Short–time preventive treatment with OM–85 protects against weight loss caused by RSV infection. Mice were treated intranasally with OM–85 (1mg) 1 day prior to RSV infection. Afterwards, mice were infected with RSV (1x10^7^ PFU/ml) and received another OM–85 boost 6h later. **(A)** Experimental design. **(B)** Percentage of weight loss relative to day 0 (right before infection). **(C)** Total cell number and differential cell counts in BAL. **(D)** RSV viral load detected in lung tissue by real–time PCR (viral copies/g of lung tissue). **(E)** Viral titer in the lung (PFUs/g lung tissue). **(F, G)** Representative images of lung tissue section stained with H&E and its respective inflammation scores. Scale bars = 200 μm. Data from 2 independent experiments (n = 6 per group). All data are expressed as mean ± SEM. Multiple groups were compared using Kruskal–Wallis, except in **(D, E)** in which the comparison between groups was made using Mann–Whitney test. *p < 0.05.

**Figure 3:**
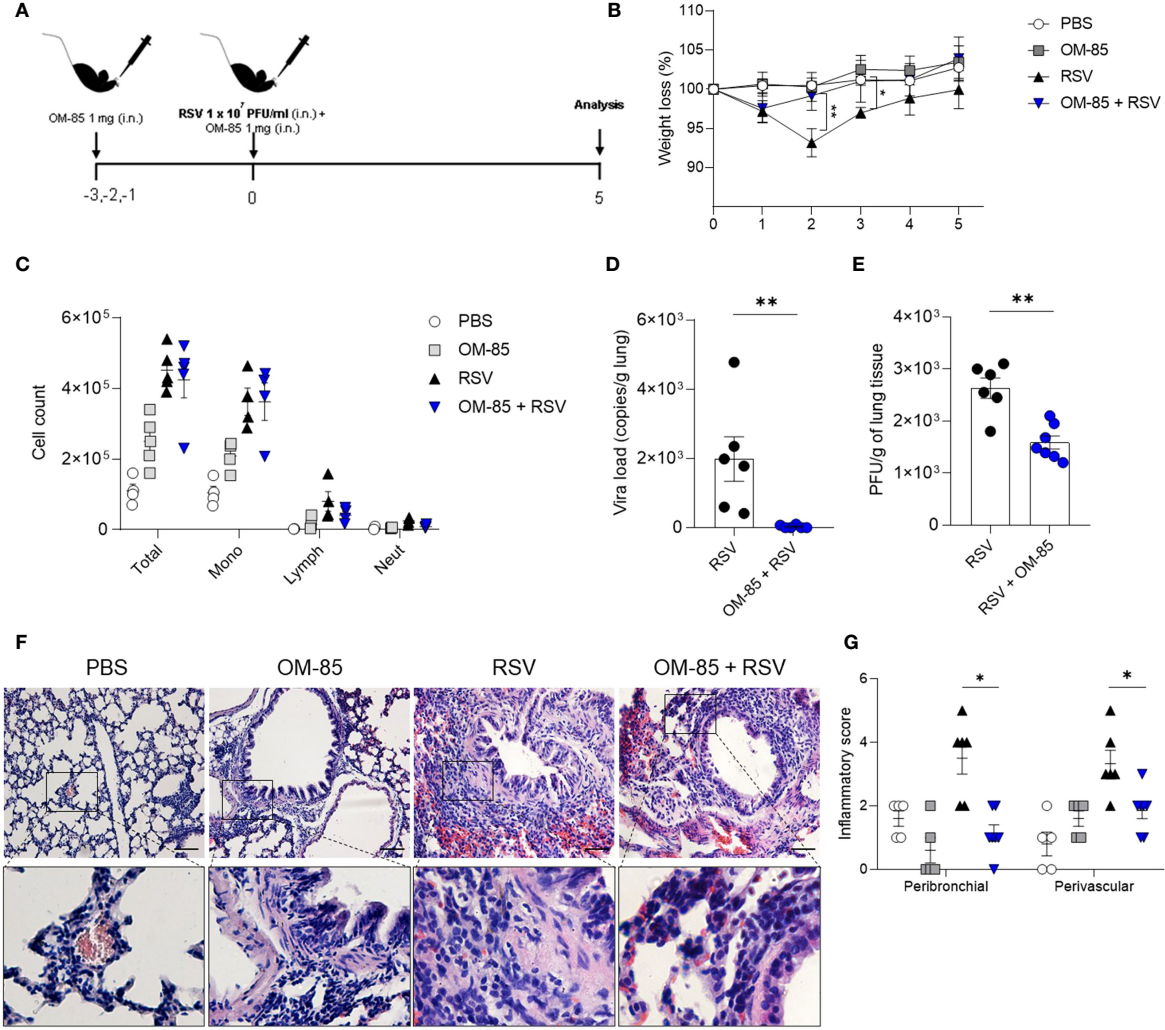
OM–85 pretreatment starting 3 days prior to infection protects against RSV–induced disease. Mice were treated intranasally with OM–85 (1mg) 3 days prior to RSV infection. Afterwards, mice were infected with RSV (1x10^7^ PFU/ml) and received another OM–85 boost 6h later. **(A)** Experimental design. **(B)** Percentage of weight loss relative to day 0 (right before infection). **(C)** Total cell number and differential cell counts in BAL. **(D)** RSV viral load detected in lung tissue by real–time PCR (viral copies/g of lung tissue). **(E)** Viral titer in the lung (PFUs/g lung tissue). **(F, G)** Representative images of lung tissue section stained with H&E and its respective inflammation scores. Scale bars = 200 μm. Data from 2 independent experiments (n = 6 per group). All data are expressed as mean ± SEM. Multiple groups were compared using Kruskal–Wallis, except in **(D, E)** in which the comparison between groups was made using Mann–Whitney test. *p < 0.05. **p < 0.01.

**Figure 4:**
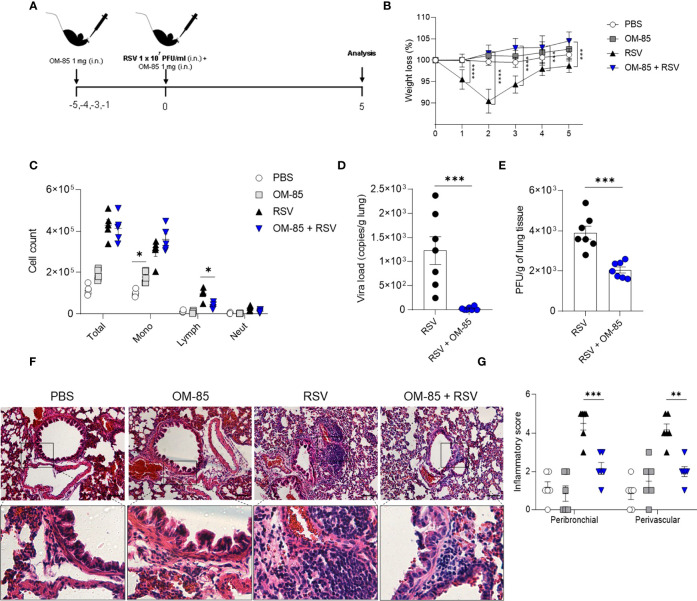
OM–85 pretreatment starting 5 days prior to infection robustly confers protection and viral clearance during RSV–induced disease. Mice were treated intranasally with OM–85 (1mg) 5 days prior to RSV infection (four OM–85 delivery). Afterwards, mice were infected with RSV (1x10^7^ PFU/ml) and received another OM–85 boost 6h later. **(A)** Experimental design. **(B)** Percentage of weight loss relative to day 0 (right before infection). **(C)** Total cell number and differential cell counts in BAL. **(D)** RSV viral load detected in lung tissue by real–time PCR (viral copies/g of lung tissue). **(E)** Viral titer in the lung (PFUs/g lung tissue). **(F, G)** Representative images of lung tissue section stained with H&E and its respective inflammation scores. Scale bars = 200 μm. Data from 3 independent experiments (n = 8 per group). All data are expressed as mean ± SEM. Multiple groups were compared using Kruskal–Wallis, except in **(D, E)** in which the comparison between groups was made using Mann–Whitney test. *p < 0.05. **p < 0.01, ***p < 0.001, ****p < 0.0001.

**Figure 5:**
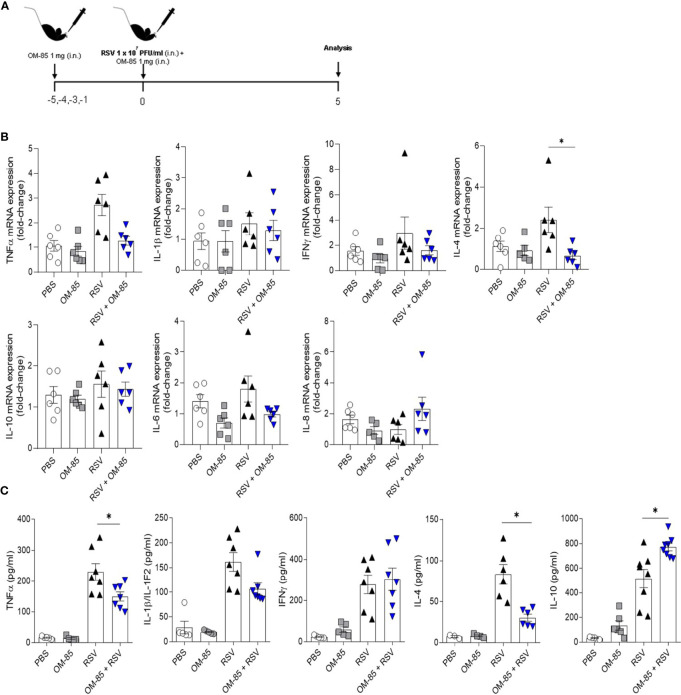
Preventive treatment with OM–85 reduces the pro–inflammatory cytokines production due RSV infection in the lungs. Mice were treated intranasally with OM–85 (1mg) 5 days prior to RSV infection (four OM–85 delivery). Afterwards, mice were infected with RSV (1x10^7^ PFU/ml) and received another OM–85 boost 6h later. BAL and lung were harvested at day 5 post–infection. **(A)** Experimental design. **(B)** *Tnfa, Il1b, Ifng, Il4, Il10, Il6* and *Cxcl15* (IL–8) gene expression in the lungs detected by real–time PCR (fold change compared to untreated/uninfected control). **(C)** Production of TNFα, IL–1β, IFNγ, IL–4 and IL–10 in the lungs measured by ELISA. All data are expressed as mean ± SEM. Multiple groups were compared using Kruskal–Wallis. *p < 0.05.

OM-85 has a protective effect against overall upper and lower airways respiratory infections and asthma both in animal and human studies. In the current study we investigate whether intranasal OM-85 treatment given prior to RSV infection alters favourably disease outcomes. Mice were exposed to the same concentration of OM-85 intranasal treatment used in the therapeutic regiment, but this time given one day prior to infection and at the same day 6 hours later (**Figure 2A**). We found that the treatment protected against weight loss on day 2 post-infection, maintaining the weight of animals slightly above that of animals that did not receive treatment until day 5 post-infection (**Figure 2B**). However, we did not observe any changes in cell recruitment to lung (**Figure 2C**) nor in the viral loads (**Figures 2D, E**) despite a trend towards decrease in viral load. Similarly, OM-85 did not protect from lung perivascular and peribronchial inflammation (**Figures 2F, G**). Proinflammatory and anti-inflammatory production in the lung was not affected by the treatment (**Figure S2**). Together these data suggested that a preventive treatment with OM-85 may need longer exposure to provide a robust protection against RSV infection. In a follow-up study we now treated mice for 3 days prior to infection (**Figure 3A**) and found that this regiment protected against weight loss at days 2 and 3 post-infection (**Figure 3B**). Although we did not observe any changes in cellular infiltrate in the lungs (**Figure 3C**) we did observe a significant statistical reduction in the viral loads in the group treated with OM-85 (**Figures 3D, E**). Consistently, OM-85 treatment decreased the perivascular and peribronchial inflammation in the lung (**Figures 3F, G**). This protective environment impaired IL-4 production in the lungs, a pro-inflammatory cytokine associated with the severity of the disease caused by RSV but did not modulate other cytokines (**Figure S3**). Considering the regiment-dependent progressive improvement of its protective effect, we investigated whether a longer exposure to OM-85 pre-treatment would conserve this trend and provide a greater protective response against the virus. Mice were then exposed to 5 days of OM-85 treatment prior to infection with RSV (**Figure 4A**). Animals which received the treatment had reduced weight loss starting day 1 post infection (**Figure 4B**). This time, OM-85 treatment not only reduced cellular infiltrate into the lungs (**Figure 4C**), but also reduced viral expression and replication in the airways (**Figures 4D, E**). Compared to untreated mice, there were also significant less inflammatory cells and structural damages in the lungs of treated mice (**Figures 4F, G**). Contrasting to what was found shorter pre-treatment periods, early-prolonged OM-85 pre-treatment significantly and efficiently prevented production of IL-4 (mRNA and protein) and TNFα after RSV infection and increased anti-inflammatory cytokine IL-10 production at protein levels in the lungs (**Figures 5A**–**C**). To be able to identify the minimum dose needed to a protective effect of OM-85 against RSV-induced disease, we lowered the treatment doses to 500 µg and 100 µg of OM-85. We observed that using 100 µg there was no protection against the disease, in contrast to the 500 µg which protected the mice well from the inflammation caused by RSV, although this effect was not as robust as seen with 1 mg (**Figure S4**). We have then decided to continue with the experiments with a 1 mg dosage. Overall, these results support that long time a minimum of 5 days of preventive exposure to intranasal administrated OM-85 completely abrogate ameliorates viral-induced disease in response to RSV infection in these experimental conditions.

### OM-85 Acts as Immunomodulator During RSV-Induced Disease

**Figure 6:**
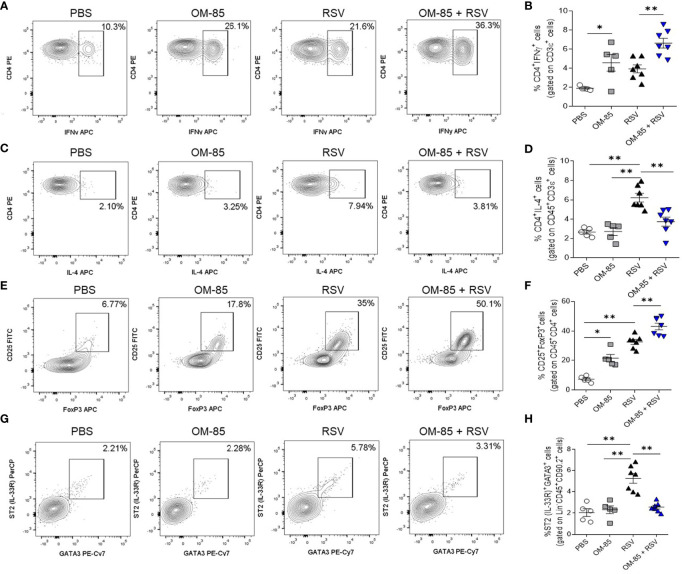
Preventive treatment with OM–85 modulates lymphocytes and lymphoid cell types in the lung in response to RSV infection. Mice were treated intranasally with OM–85 (1mg) 5 days prior to RSV infection (four OM–85 delivery). Afterwards, mice were infected with RSV (1x10^7^ PFU/ml) and received another OM–85 boost 6h later. BAL and lungs were harvested at day 5 post–infection. **(A, B)** Percentage of CD3^+^CD4^+^IFNγ^+^ Th1 cells in the lung and its representative FACS profile. **(C, D)** Percentage of CD45^+^CD3^+^CD4^+^IL–4^+^ Th2 cells in the lung and its representative FACS profile. **(E, F)** Percentage of CD45^+^CD4^+^CD25^+^FoxP3^+^ Treg cells in the lung and its representative FACS profile. **(G, H)** Percentage of Lineage^–^CD45^+^CD90.2^+^ST2^+^Gata3^+^ ILC2 cells in the lung and its representative FACS profile. All data are expressed as mean ± SEM. Multiple groups were compared using Kruskal–Wallis. *p < 0.05. **p < 0.01.

To further explore the efficacy of OM-85 in suppressing RSV induced airway inflammation *in vivo*, we used FACS analysis to address the effect of an early 5-day preventive OM-85 intervention on cellular inflammatory profile in the lungs. As RSV infection is known for skewing the Th1/Th2 cytokine balance toward increased levels of Th2 cytokines and reduced Th1 profile (40), we first investigated these T cell phenotypes. OM-85 treatment significantly improved pulmonary Th1 cell population, even without virus presence (**Figures 6A, B**). Th2-biased profile was decreased when compared to untreated mice (**Figures 6C, D**), suggesting a shift between Th1/Th2 cell population. Like what was found with a Th1 population, significant expansion of Treg FoxP3+ cells in the lung were observed in OM-85-treated animals even without infection (**Figures 6E, F**). Moreover, RSV infection leads to activation of group 2 innate lymphoid cells (ILC2) which is known to be associated with more severe RSV-bronchiolitis (41, 42). We also observed that OM-85 prevented the increase of ILC2 percentages (Thy 1.2^+^ Lineage^-^, ST2^+^ Gata3^+^) in the lungs, keeping it at basal levels (**Figures 6G, H**).

**Figure 7:**
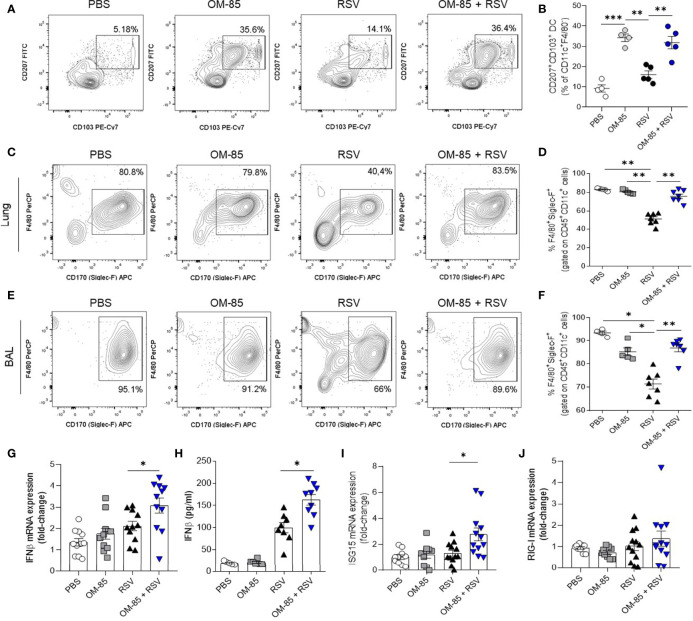
Preventive treatment with OM–85 expands dendritic cells and alveolar macrophages and contributes do antiviral response improvement. Mice were treated intranasally with OM–85 (1mg) 5 days prior to RSV infection (four OM–85 delivery). Afterwards, mice were infected with RSV (1x10^7^ PFU/ml) and received another OM–85 boost 6h later. BAL and lungs were harvested at day 5 post–infection. **(A, B)** Percentage of CD11c^+^F4/80^–^CD207^+^CD103^+^ conventional dendritic cells in the lung and its representative FACS profile. **(C–F)** Percentage of ^+^CD11c^+^F4/80^+^Siglec–F^+^ alveolar macrophages in lung and BAL, and its representative FACS profile. **(G)** *Ifnb1* gene expression in the lungs detected by real–time PCR (fold change compared to untreated/uninfected control). **(H)** Production of IFNβ in the lungs measured by ELISA. **(I, J)** *Isg15* and *Ddx58* (RIG–I gene) gene expression in the lungs detected by real–time PCR (fold change compared to untreated/uninfected control). All data are expressed as mean ± SEM. Multiple groups were compared using Kruskal–Wallis. *p < 0.05. **p < 0.01, ***p < 0.001.

As OM-85 intranasal treatment has been shown to modulate dendritic cells towards a more tolerogenic profile in experimental asthma (37) we also analyzed this population in our model. We found that populations of tolerogenic CD207^+^CD103^+^ DCs were significantly increased in OM-85 + RSV and RSV-infected mice (**Figures 7A** **, B**). Interestingly, OM-85 alone was also able to increase this population (**Figures 7A** **, B**), suggesting that OM-85 was reprogramming these DCs to promote less Th2 cell-subtype activation.

It is known that alveolar macrophages (AMs) play an important role in controlling RSV infection (43, 44). Lung-resident AMs are mainly characterized by their specific surface marker CD170 (Siglec-F) which is not expressed by interstitial or inflammatory macrophages. We found that OM-85 treatment maintained a high percentage of AMs compared to untreated animals (**Figures 7C**–**F**). These results clearly demonstrate that OM-85 prevents RSV-induced inflammatory response in the airways.

To better understand the protective role of OM-85 against RSV in this regiment, we decided to investigate the antiviral response pathway, since OM-85 is recognized as an immunomodulator of IFNβ production (32). We found that OM-85 increased IFNβ expression and production in the lungs even at day 5 post infection (**Figures 7G, H**). Although the treatment did not modulate RIG-I expression, it did boost the expression of ISG15, an important ISG (**Figures 7I, J**). Thus, these data suggest a broad protective effect of OM-85 when administered intranasally in RSV-infected mice, modulating an array of immune cells in the lung, both from innate and adaptative pathways, also improving antiviral defenses mediated by type 1 interferon.

### Antiviral Effect of OM-85 Relies on Type I Interferon Pathway

**Figure 8:**
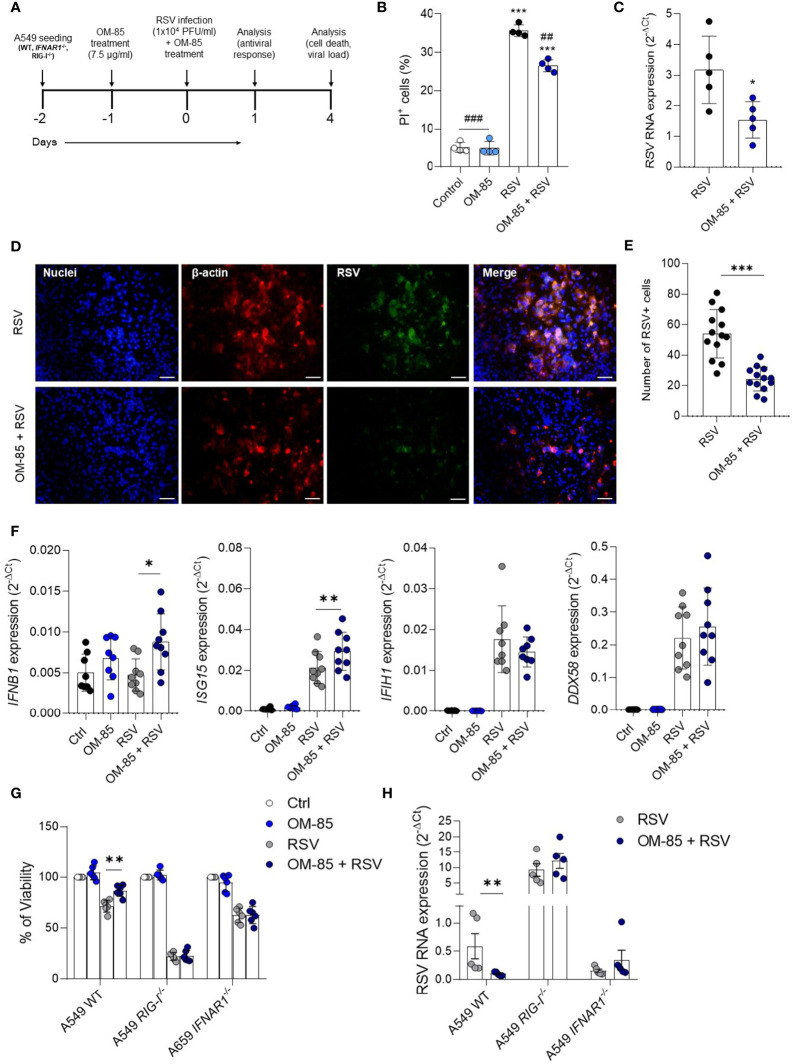
Pretreatment with OM–85 protects against RSV infection *in vitro* depending on type I interferon pathway. Mycoplasma–free A549 cells (8 x 10^4^ cells/ml) were treated with OM–85 (7.5 ug) for 24hs. Afterward cells were washed and placed fresh new media and then infected with RSV (10^4^ PFU/ml) and received a boost of OM–85 treatment. The culture was left for 1 [as in **(F)** or 4 days (as in **(B–D, E, G, H)**]. **(A)** Experimental design. **(B)** Percent of PI (propidium iodide) positive cells detected by flow cytometry. **(C)** RSV RNA levels detected using real–time PCR (2^–ΔCt^). **(D)** Immunofluorescence images showing staining of cell nuclei (Hoescht – blue), B–actin (red) and RSV (green). Scale bars = 0.5 μm. **(E)** Number of RSV+ cells measured in the immunofluorescence images. **(F)** *IFNB1*, *ISG15*, *IFIH1* (MDA5), and *DDX58* (RIG–I) gene expression detected by real–time PCR. **(G, H)** A549 WT, A549 IFNAR1^–/–^, and A549 RIG–I^–/–^ cells (8 x 10^4^ cells/ml) were treated with OM–85 (7.5 ug) for 24hs. Afterward cells were washed and placed fresh new media and then infected with RSV (10^4^ PFU/ml) and received a boost of OM–85 treatment. The culture was left for 4 days until analysis. **(G)** Cell viability assessed by MTT assay using untreated/uninfected control as 100% of viability. **(H)** RSV RNA levels detected using real–time PCR (2^–ΔCt^). All data are expressed as mean ± SEM. Multiple groups were compared using Kruskal–Wallis, except in **(C, E)** in which the comparison between groups was made using Mann–Whitney test. # indicates the statistical difference compared to RSV. ##*p* < 0.01, ###*p* < 0.001. *p < 0.05. **p < 0.01, ***p < 0.001.

To further explore the significance of these latter findings on antiviral response, we experimented with human airway epithelial cells, a preeminent natural RSV target and important IFNβ producers. We first set the optimal OM-85 concentration for A549 cell line which does not cause cell death (**Figure S5**). Cells were then pre-treated with OM-85 (7.5 µg/ml) for 24 hours and then infected with RSV followed by a boost of OM-85 treatment. Analysis was made at different timepoints post infection (**Figure 8A**). OM-85 treatment protected against cell death caused by infection and significantly reduced RSV-RNA expression in those cells (**Figures 8B, C**). Accordingly, there was significant fewer RSV within the cells treated with OM-85 (**Figures 8D, E**). We observed that as seen in the lung, OM-85 increased IFNβ and ISG15 expression following RSV infection (**Figure 8F**). To confirm these findings related to the antiviral response, we investigated the effects of OM-85 in A549 cells lacking the type I response pathway (IFNAR and RIG-I knockout cells). We found that the protective role of OM-85 was completely abolished in the absence of genes related to the type I interferon response (**Figures 8G, H**), supporting the idea that the anti-viral effects demonstrated by OM-85 in various examples (32–35, 38, 39, 45, 46) including in this study using RSV as infective agent may be at least partially acting through modulation of the interferon response.

## Discussion

In recent years, studies have suggested an important role of bacterial lysates in maintaining respiratory antiviral immunity through modulation of the immune response (47). Among them, studies demonstrating that OM-85 a bacterial lysate obtained from human airway–derived bacterial strains, effectively protects against several viruses in human cells and animal models. This led us to investigate if this product would also parallel our recent work where therapeutic intake of orally given microbiota-derived acetate was shown to confer protection against RSV in therapeutic settings. Considering the former and recent work evidencing superior protection of OM-85 against various experimental asthma models when administrated intranasally, we asked whether OM-85 directly used in the airways can prevent from viral infection using our RSV mouse model. Our study demonstrates that early-prolonged use of intranasal administration of OM-85 effectively protects against specific RSV-induced disease, preventing viral replication and associated inflammation sequelae in the lungs. Several hallmarks can be concluded from this study. At first, the protective effect of OM-85 failed to recapitulate acetate results in therapeutic settings. Secondly, when used as preventive measure, OM-85 conferred progressive protection against RSV reaching full protection after 4 intranasal intakes in 5 days. In these experimental conditions, OM-85 efficacy seems to be directly associated with the time of exposure to treatment prior to infection. Pre-treatment with only 1 day or 3 days with 1 mg of active principal ingredient was progressively demonstrating protection (viral load and body weight loss) with a maximum efficacy after 5 days (4 intranasal intakes of OM-85 of 1 mg each) prior to infection. Assessing the dosing in this best regimen of 5 days, an increasing concentration of 0,1 mg and 0,5 mg conferred some and good protection respectively. Investigating further inflammatory parameters such as lung inflammatory endotypes as associated measure of efficacy demonstrated that only the 1 mg dose given 4 times in 5 days completely abrogated RSV infection and all associated lung inflammatory sequelae including but not limited to the prevention of peribronchial and perivascular inflammation in the lungs.

**Figure 9:**
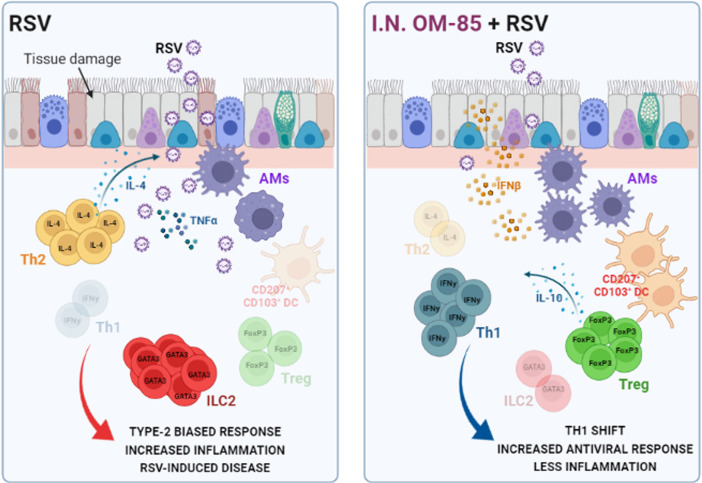
Preventive effects of airway–delivered OM–85 during RSV infection. Preventive treatment with OM–85 directly delivered into the airways promote a pro–resolutive homeostatic environment in the lung mucosa, increasing alveolar macrophages, tolerogenic DCs, Treg, and Th1 cell population in locus. This profile enhances viral clearance, increasing IFNβ production, preventing Th2–biased response and ILC2–caused hyperresponsiveness in the lung, ameliorating RSV–induced disease. Image created with https://biorender.com/.

These data indicate that OM-85 mechanism of protection against RSV infection required time, even minimal, to completely modulate the protective immune response monitored by lung virus load, inflammatory cell content and body weight. A summary scheme of our findings is proposed in **Figure 9**.

To the best of our knowledge, this the first study with OM-85 used directly into the airways to evaluate protection against viral respiratory infection. Thus, paralleling recent work using the same route but to prevent asthma (37). Thirdly, as in experimental asthma, a dose of 1mg conferred full protection, however here, with a shorter regimen as only 5 days were sufficient. This is contrasting with most studies performed *in vivo* with OM-85 using numerous disease animal models where changes of oral dosing and/or regimens failed demonstrating difference in efficacy. Considering the importance of pharmacodynamic parameters used for clinical trial design, these data are likely to be of high translational value to set dosing and regimen for this new route of administration. Fourthly, this intranasal preventive regimen confers protection specifically against RSV, similar to what is observed with oral administration of the OM-85 bacterial lysate preparation widely used to prevent recurrent upper respiratory infections in human trials (48, 49).

While these results share some common mechanistic features from our previous findings on improving type 1 interferon responses and increasing different interferon-stimulated gene expression to defeat RSV, they are different. On the one hand, the therapeutic treatment used with acetate directly delivered into the lung protected against RSV infection by modulating the type I interferon response (30), while here, this effect took place when OM-85 was used preventively. Secondly, this *in vivo* study brings a fourth virus by which OM-85 can protect against infection (after H1N1, hRV and SARS-CoV-2) and as such, consolidate the general concept that this product of bacterial origin exerts strong potency against respiratory viruses when used as preventive measure.

As demonstrated, treatment with OM-85 mediated an IFNβ response in both *in vivo* and *in vitro* experiments, in sync with other studies performed with the oral route or *in vitro* settings (32, 36, 37, 39), but this does not limit its antiviral mechanism against different viruses (33, 35, 39). In the present study, we found that IFNβ production in cells treated with OM–85 occurs only after virus infection. While this can be attributed to two cell types (A549 vs. primary DCs), the antiviral effect induced by OM–85 is probably armed at the regulatory cell level as pre–alert state (32, 33), and/or upon host need. Other studies have analysed the differential gene expression mediated by OM–85 treatment and found that several pathways are modulated by OM–85, including that of metabolism (37). One hypothesis could be that OM–85 is reprograming these cells through epigenetic modification activity.

RSV can evade antiviral response by inhibiting RIG–I activation through its non–structural proteins (50). Induction of RIG–I–dependent IFN–I is important to strengthen antiviral response against RSV (51, 52). Although we did not find that OM–85 activates RIG–I during RSV infection, the protective mechanism was completely lost in the absence of either RIG–I or IFNAR1 molecules, suggesting that OM–85 somehow relies on this activation pathway to exert its protective role *in vitro*. In that line, it has been shown that intranasally administered *Lactobacillus rhamnosus* strains modulates the TLR3/RIG–I–triggered antiviral respiratory immune response against RSV by increasing IFN–α, IFN–β, and IL–10 production in the lung (53). We did not find RIG–I modulation in the *in vivo* experiments. Nevertheless, the ideal time to observe the alteration on this protein would be even prior to 5 days after infection, which we did not look at. Although OM–85 is modulating the IFN–I response *in vivo*, as mentioned above, it is possible that other mechanisms are involved in the protection against RSV disease. Fourthly and considering the former, *in vivo* studies revealed that treatment by OM–85 might be important to balance of the inflammatory and adaptive response during infection leading to the amelioration of the disease. This cell modulation by OM–85 was exemplified by other parameters of the immune response, such as ILC2, consolidating its effect on this same intranasal route in asthma models (37), as well as with T helper lymphocytes and alveolar macrophages suggesting a faster IFN response towards a viral challenge.

Along these lines, acute bronchiolitis caused by RSV infection is known to activate Th2 cell–related cytokines such as IL–4 and IL–5, resulting in an imbalanced immune response leading to airway mucus overproduction (41). This is likely an important pathway linking RSV and asthma (54). Remarkably, OM–85 treatment prevented a Th2–biased response caused by RSV infection in the lungs, restoring the Th1/Th2 imbalance. In line with these findings, some studies have shown that OM–85 promotes immune system maturation in children *via* downregulation of Th2 through activation of Treg cells (55). Through this pro–regulatory effect of OM–85 treatment, there was also improved differentiation of FoxP3+ Treg cells in the lungs, which are important for the immune response regulation and viral clearance. It is important to note that OM–85 facilitated the recruitment of Th1 and Treg cells in the lungs even in the absence of RSV. One possible explanation is that, among the various immune cells, the microbiota has been shown to be associated with the development of Th1 and Treg cells to maintain tissue homeostasis (56, 57). As the manufacturing process of OM–85 is generating a microbial product comprising chemical moieties which conferred protection in several disease model including those from our study, one could speculate that they would mimic microbiota metabolites and/or influence the anti–viral and anti–inflammatory immunity illustrated with RSV. Example of such immunoregulation and protection involving also Treg cells has been characterized *in vivo* in asthma as disease model (37, 58). Accordingly, this safe degradation microbial product may also comprise metabolite which may mimic some present from the gut.

Additional evidence of these antiviral and inflammatory pathways activated by OM–85 in this RSV model is that increased levels of ILC2 were found in nasal aspirates of hospitalized infants with more severe RSV–bronchiolitis (42). RSV driven IL–33–activated ILC2 is crucial for the development of airway inflammation, including eosinophilia and airway hyperreactive response, through ILC2–specific IL–13 production (59). Interestingly, in our study OM–85 treatment prevented the enhancement of ILC2 ST2+ in the lungs. Accordingly, airway administration of OM–85 was found to block experimental asthma by targeting dendritic cells and ILC2 (37). Although we have not measured IL–33, a cytokine associated with enhanced airway responsiveness/inflammation, the lung immunopathology was prominently mild in the animals treated with OM–85. ILC2 inhibition improves acute bronchiolitis induced by RSV (60), and IFN–I is involved in restricting ILC2 immunopathology (61). This suggests that the production of IFNβ by OM–85 may be limiting ILC2 expansion during RSV infection.

Considering the importance of endotype cells in the mounting of the immune response, dendritic cells play a central role in the induction of the RSV–specific adaptive immune response. RSV infection activates DCs and initiate the T–cell response (62). Studies have shown that CD103^+^ conventional DCs (cDCs) are important for viral clearance and protective immunity against RSV infection (63, 64). In our study we found that OM–85 expanded the population of tolerance–associated CD207/langerin^+^CD103^+^ cDCs in the lung, here again recapitulating such finding found in asthma (36). This cDC subtype has a unique capacity to induce and activate FoxP3^+^ Treg cells to help control an exacerbated immune response (65). Since we also found that OM–85 is expanding FoxP3+ Treg cells in the lung, the cDCs likely promote a tolerogenic landscape after OM–85 treatment contributing to a faster resolution of RSV–induced disease. Considering this protective expansion of tolerogenic cells in the lung is comparable in OM–85 and OM–85–RSV arms, it is likely that this additional anti–viral response is more general than RSV–specific, thus confirming its preventive use as general anti–viral, innate non–specific drug (33–35, 37, 38, 47).

Importantly, macrophage polarization is also likely involved in RSV infection (66, 67). Although there is evidence showing that alveolar macrophages necroptosis drives disease pathogenesis during RSV infection (68), there are also evidence indicating that AM can control/attenuate RSV pathology (44, 68–71). Our study showed that OM–85 treatment used as preventive measure consistently maintained AM population in the lungs, while RSV–infected untreated animals presented a pronounced reduction in these cells. This decrease in AM population can be partially explained by previous evidence demonstrating that RSV elicits MMP–12–producing macrophages over AM (72). OM–85 prevents this shift contributing to controlled RSV–induced disease. Investigating other macrophage profiles is crucial to better understand the role of OM–85 towards these cells during RSV infection, which we did not include in this study, but must be further explored. As one of the main producers of IFNβ during RSV infection is the AM (70), this may be a key player explaining the different mechanism of action of OM–85 in the *in vivo* and *in vitro* approaches. Although we did not measure IFNβ production specifically in lung epithelial cells, in the *in vitro* experiments there was no immune cells involvement such as AM, but only respiratory epithelial cells. *In vitro*, OM–85 is modulating IFN–I pathway to defend against RSV infection, as it has been shown to be happening against other viruses (32, 46). However, *in vivo*, OM–85 acts in a more complex way, involving cell types other than IFNβ–producing cells, such as alveolar macrophages and/or epithelial cells. As such, our findings contrast with a study where intranasal administration of *Bifidobacterium longum* protects against viral–induced lung inflammation and injury during influenza infection without modulating type I interferons in the airways (73).

In summary, airway–administered OM–85 was highly efficient against RSV infection, especially when given for at least 5 days prior to infection using 1 mg dose of API, and this may indicate that this bacterial lysate induces diverse airway microbial stimulation and its direct contact to the airway mucosa environment contributes to regulate response to viruses. In addition, we provide evidence that OM–85 can be considered as a potential strong protective microbial product against RSV infection, when used intranasally as preventive regimen, without providing any harm to the host. Further studies are necessary to determine whether administration of this bacterial lysate to the airways can protect humans from clinically significant RSV bronchiolitis.

## Data Availability Statement

The original contributions presented in the study are included in the article/**Supplementary Material**. Further inquiries can be directed to the corresponding author.

## Ethics Statement

The animal study was reviewed and approved by CEUA/PUCRS protocol #10180.

## Author Contributions

ADS, RS, CP, and KA conceived the study and designed the experiments. KA, GC, LS, SB, JP, JBG, EN, and GR performed the *in vivo* experiments. KA, SB, VS, and GDS performed the *in vitro* analysis. KA performed the flow cytometry analysis. GC and GR performed the histopathology procedures. DM generated A549 RIG–I and IFNAR1 KO cells and contributed to data interpretation. ADS, CP, and KA wrote the manuscript. All authors contributed to the article and approved the submitted version.

## Funding

This study received funds from FAPERGS (number 20/2551–0000258–6). AS received fellowship grant from CNPq (309400/2021–0). GC and JGB received fellowship grant from CAPES (financial code 1).

## Conflict of Interest

CP is employee of OM Pharma. AS, RS, and KA received fees from OM Pharma.

The remaining authors declare that the research was conducted in the absence of any commercial or financial relationships that could be construed as a potential conflict of interest.

The authors declare that this study received funding from OM Pharma. The funder was not involved in the collection or analysis but in the study design, interpretation of data, the writing of this article or the decision to submit it for publication, all with fairness and scientific rigour.

## Publisher’s Note

All claims expressed in this article are solely those of the authors and do not necessarily represent those of their affiliated organizations, or those of the publisher, the editors and the reviewers. Any product that may be evaluated in this article, or claim that may be made by its manufacturer, is not guaranteed or endorsed by the publisher.
