# Supplementary material for: Airway Administration of Bacterial Lysate OM-85 Protects Mice Against Respiratory Syncytial Virus Infection
Source: Front Immunol. 2022 May 5;13:867022. doi: 10.3389/fimmu.2022.867022 (PMC9118194; doi:10.3389/fimmu.2022.867022)
Supplement: Supplementary file 1 [file DataSheet_1.pdf]

Supplementary Material

1 Supplementary Table

Supplementary Table 1. Gene ID description

Gene name	Gene ID	Species
Actb	Mm02619580_g1	Mouse
Tnfa	Mm00443258_m1	Mouse
Il1b	Mm00434228_m1	Mouse
Ifng	Mm01168134_m1	Mouse
Il4	Mm00445259_m1	Mouse
Il6	Mm00446190_m1	Mouse
Cxcl15 (IL-8)	Mm00441263_m1	Mouse
Il10	Mm01288386_m1	Mouse
Ifnb1	Mm00439552_s1	Mouse
Isg15	Mm01705338_s1	Mouse
Ddx58 (RIG-I)	Mm01216853_m1	Mouse
ACTB	Hs01060665_g1	Human
IFNB1	Hs01077958_s1	Human
ISG15	Hs01921425_s1	Human

IFIH1 (MDA5)	Hs00223420_m1	Human
DDX58	Hs01061436_m1	Human

2. Supplementary Figures

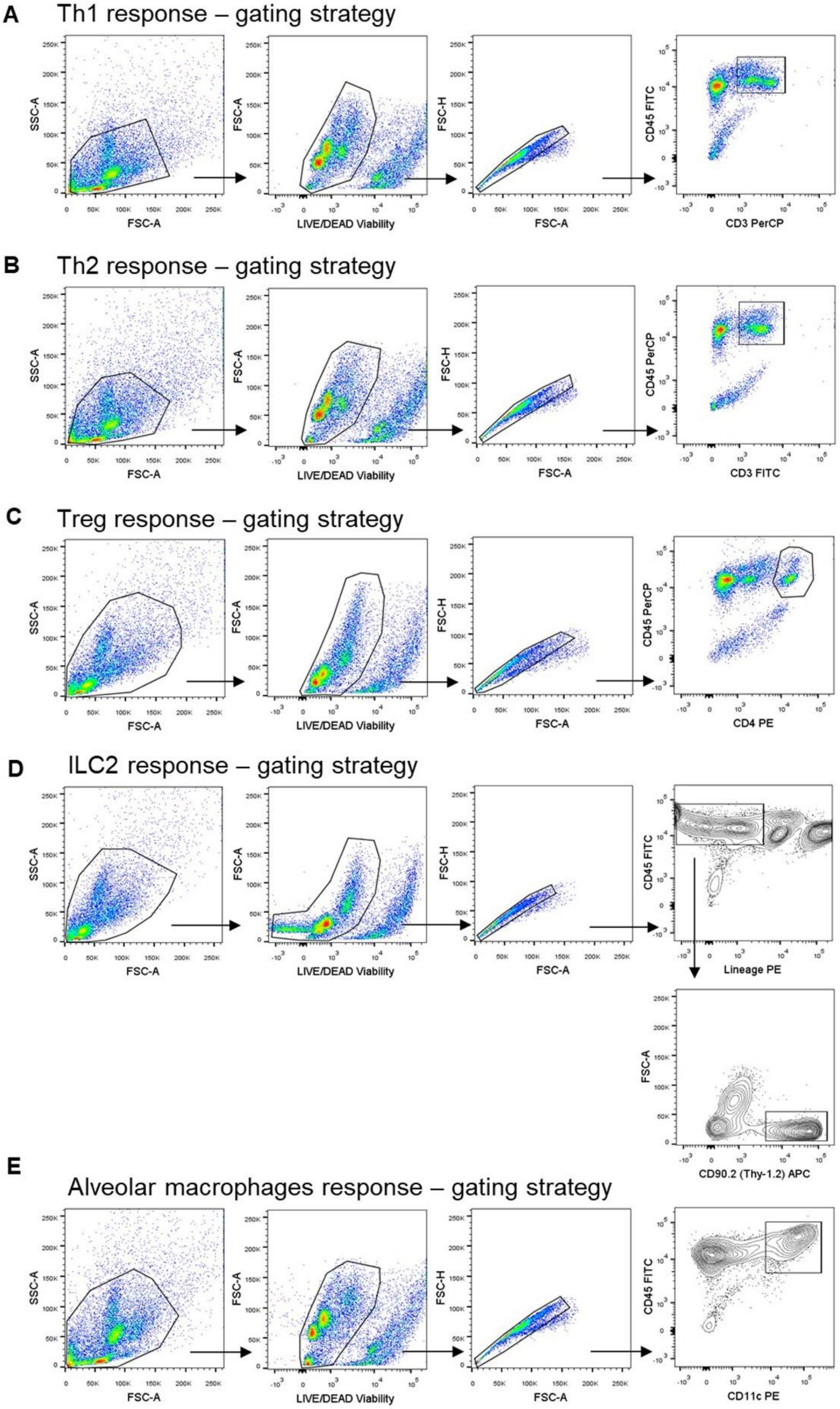

Supplementary Figure 1. Gating strategy of each immune cell population. (A) Gating strategy of Th1 lymphocytes. **(B)** Gating strategy of Th2 lymphocytes. **(C)** Gating strategy of Treg lymphocytes. **(D)** Gating strategy of ILC2. **(E)** Gating strategy of alveolar macrophages.

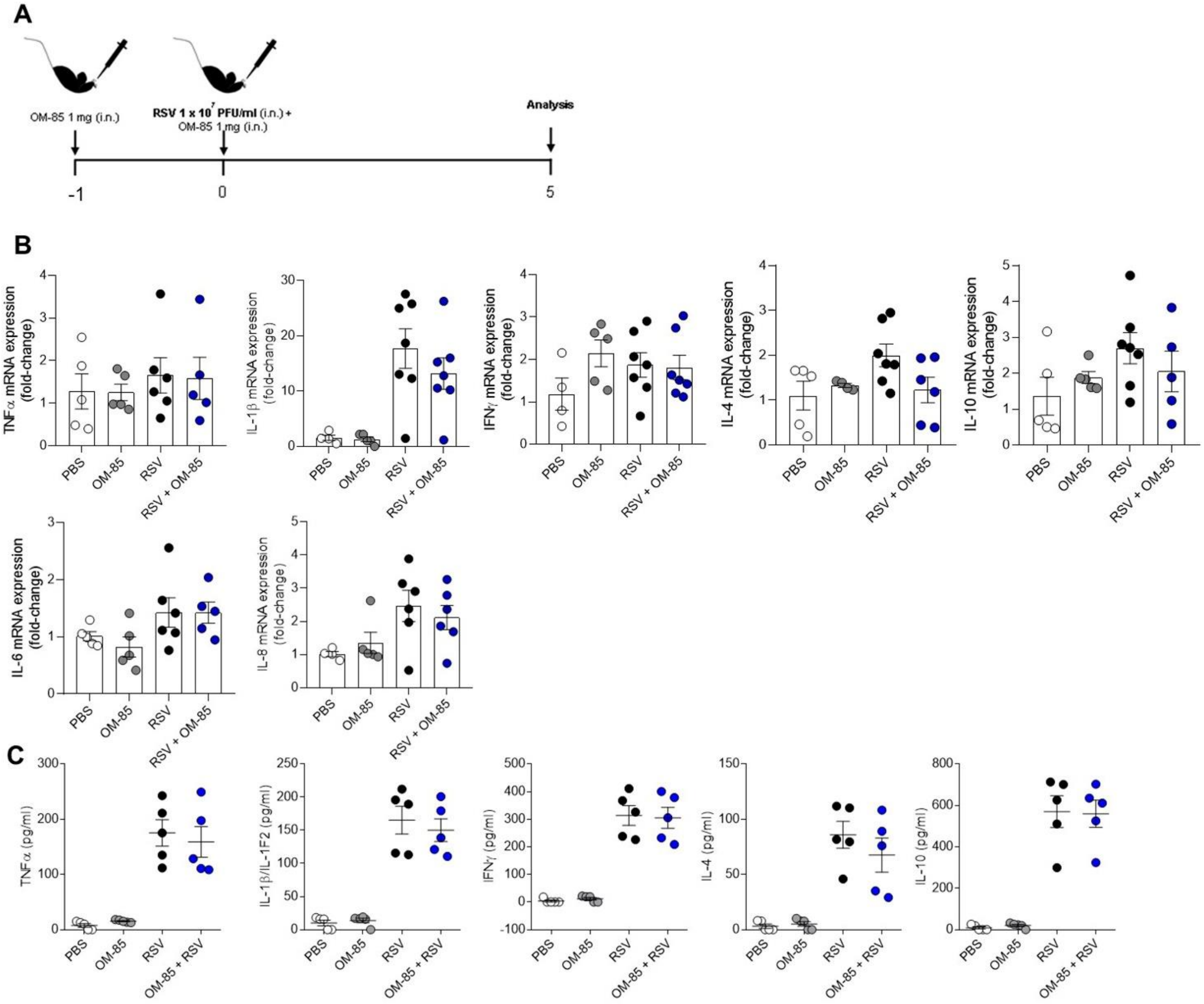

Supplementary Figure 2. Short-time preventive treatment with OM-85 does not modulate cytokine production in the lungs during RSV infection. Mice were treated intranasally with OM-85 (1mg) 1 day prior to RSV infection. Afterwards, mice were infected with RSV (1x10^7 PFU/ml) and received another OM-85 boost 6h later. BAL and lung were harvested at day 5 post-infection. **(A)** Experimental design. **(B)** *Tnfa, Il1b, Ifng, Il4, Il10, Il6* and *Cxcl15* (IL-8) gene expression in the lungs detected by real-time PCR (fold change compared to untreated/uninfected control). **(C)** Production of TNFα, IL-1β, IFNγ, IL-4 and IL-10 in the lungs measured by ELISA. All data are expressed as mean ± SEM. Multiple groups were compared using Kruskal–Wallis.

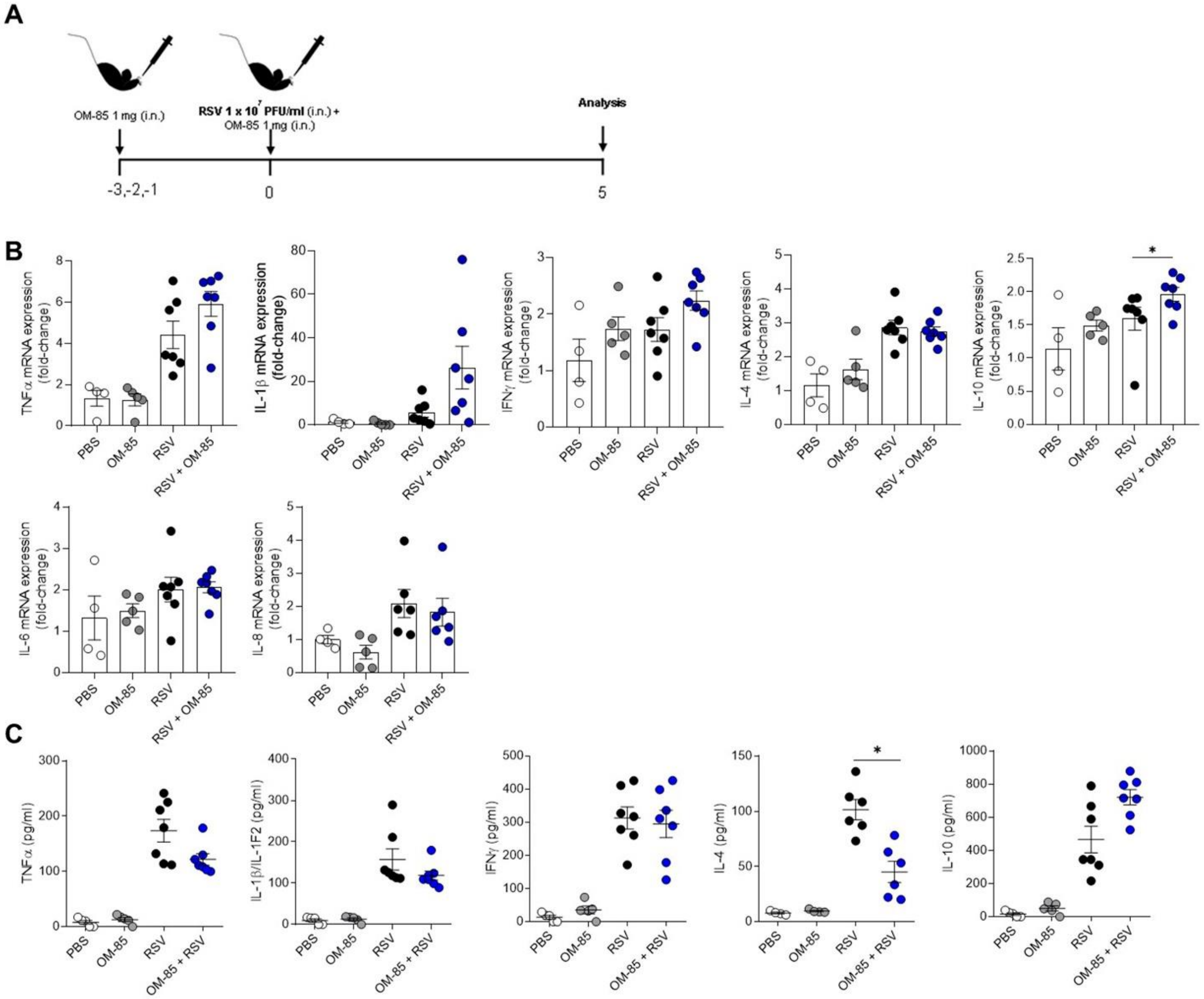

Supplementary Figure 3. OM-85 pretreatment starting 3 days prior to modulates IL-4 and IL-10 production in the lungs during RSV infection. Mice were treated intranasally with OM-85 (1mg) 3 days prior to RSV infection. Afterwards, mice were infected with RSV ($1x10^7$ PFU/ml) and received another OM-85 boost 6h later. BAL and lung were harvested at day 5 post-infection. **(A)** Experimental design. **(B)** *Tnfa, Il1b, Ifng, Il4, Il10, Il6* and *Cxcl15* (IL-8) gene expression in the lungs detected by real-time PCR (fold change compared to untreated/uninfected control). **(C)** Production of TNFα, IL-1β, IFNγ, IL-4 and IL-10 in the lungs measured by ELISA. All data are expressed as mean ± SEM. Multiple groups were compared using Kruskal–Wallis. $*p < 0.05$.

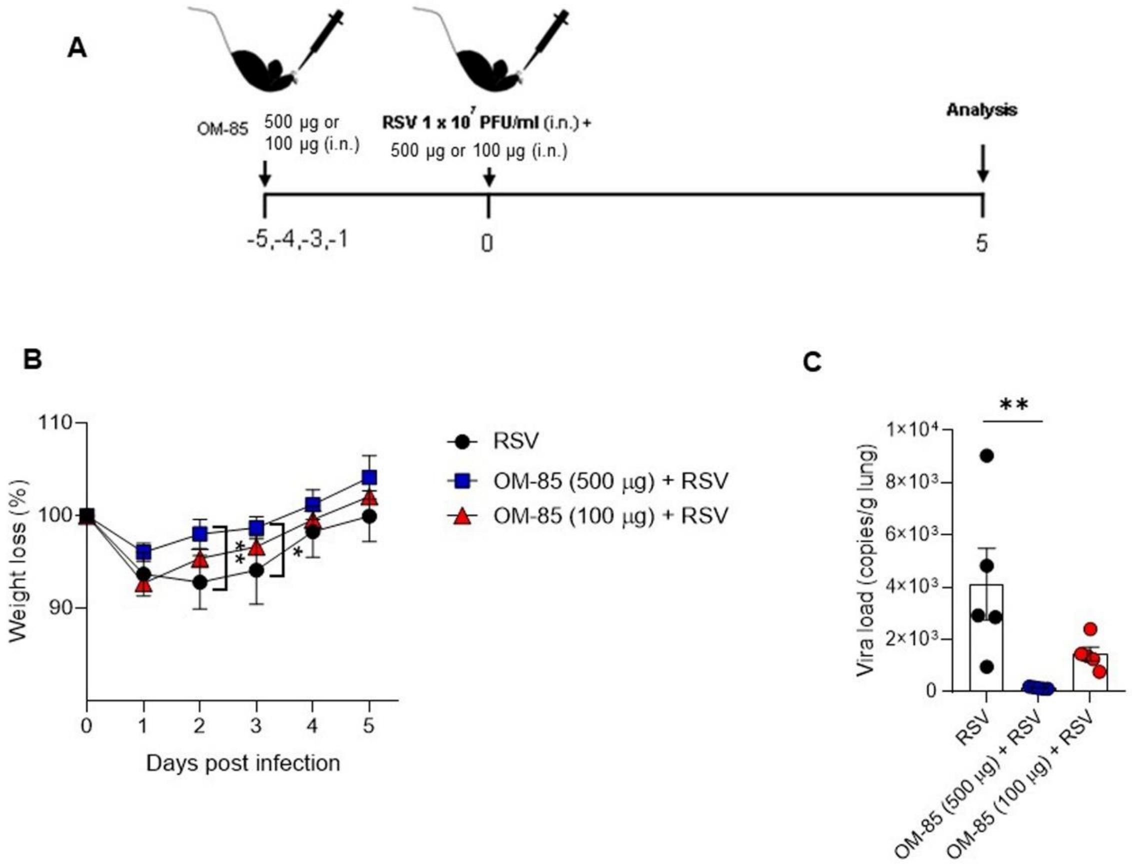

Supplementary Figure 4. OM-85 pretreatment protects against RSV infection in a dose-dependent manner. Mice were treated intranasally with OM-85 (1mg) 5 days prior to RSV infection. Afterwards, mice were infected with RSV (1x10^7 PFU/ml) and received another OM-85 boost 6h later. **(A)** Experimental design. **(B)** Percentage of weight loss relative to day 0 (right before infection). **(C)** RSV viral load detected in lung tissue by real-time PCR (viral copies/g of lung tissue). All data are expressed as mean ± SEM. Multiple groups were compared using Kruskal–Wallis. $^{**}p < 0.01$, $^{***}p < 0.001$.

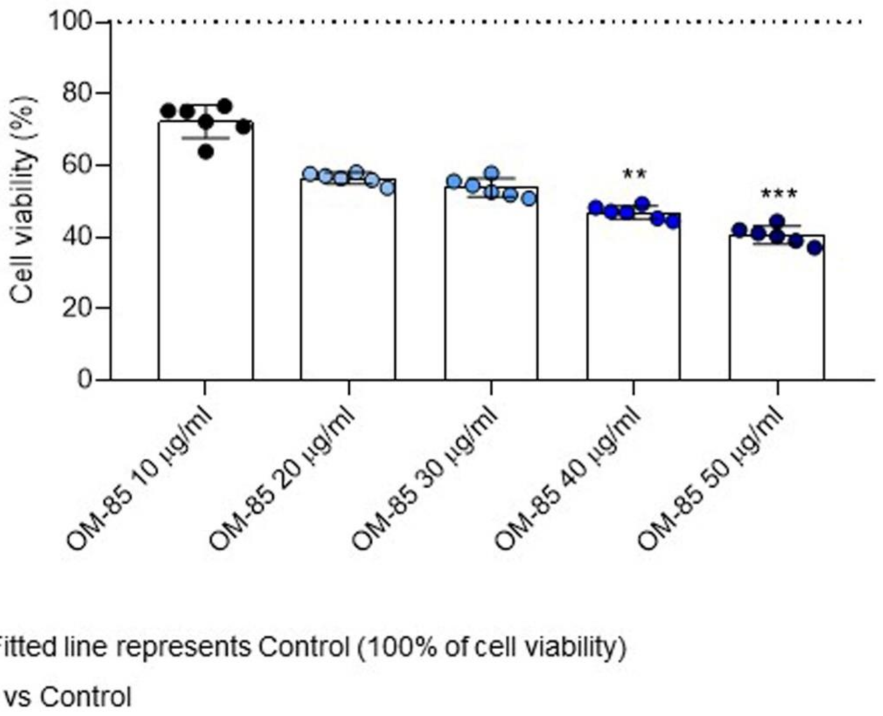

Supplementary Figure 5. *In vitro* OM-85 toxicity assay. Mycoplasma-free A549 cells (8 x 10^4 cells/ml) were treated with different concentrations of OM-85 for 96h. Cell viability was assessed by MTT assay using untreated control as 100% of viability. All data are expressed as mean ± SEM. Multiple groups were compared using Kruskal–Wallis. $**p < 0.01$, $***p < 0.001$.
